# Investigation of the acoustic emission and fractal characteristics of coal with varying water contents during uniaxial compression failure

**DOI:** 10.1038/s41598-023-29473-4

**Published:** 2023-02-08

**Authors:** Muhammad Ali, Enyuan Wang, Zhonghui Li, Naseer Muhammad Khan, Mohanad Muayad Sabri Sabri, Barkat Ullah

**Affiliations:** 1 School of Safety Engineering, China University of Mining and Technology, Xuzhou, 221116 China; 2 Department of Mining Engineering, Engineering and Management Sciences, Balochistan University of Information Technology, Quetta, Pakistan; 3 Key Laboratory of Gas and Fire Control for Coal Mines of Ministry of Education, China University of Mining and Technology, Xuzhou, 221116 China; 4 Department of Sustainable Advanced Geomechanical Engineering, Military College of Engineering, National University of Sciences and Technology, Risalpur, 23200 Pakistan; 5 Peter the Great St. Petersburg Polytechnic University, St. Petersburg, 195220 Russia; 6 School of Resources and Safety Engineering, Central South University, Changsha, 410083 China

**Keywords:** Environmental sciences, Natural hazards, Engineering

## Abstract

To investigate the effect of water on the mechanical properties and acoustic emission (AE) characteristics of coal in the failure and deformation processes. Coal samples of different content were subjected to uniaxial compression tests and AE signals were monitored. The characteristics of the AE signals were further analyzed using fractal analysis. The results show that saturated coal samples have substantially reduced mechanical properties such as uniaxial compressive strength (UCS), dissipation energy, peak stress, and elastic modulus. Under loading, stress–strain curves are characterized by five distinct stages: (1) compaction; (2) linear elastic; (3) crack stable propagation; (4) crack accelerating propagation; and (5) post-peak and residual stages. Using phase-space theory, a novel Grassberger Procaccia (GP) algorithm was utilized to find the AE fractal characteristics of coal samples in different stages. It is significant to note that AE energy does not exhibit fractal characteristics in either the first or second stages. Contrary to the first two stages, the third stage showed obvious fractal characteristics. Fractal analysis of AE time sequences indicates that fractal dimension values change as stress increases, indicating the initiation of complex microcracks in coal. In the fourth stage, the fractal dimension rapidly declines as the strength reaches its limit, indicating the occurrence of macrocracks. However, fractal dimensions continued to decrease further or increased slightly in the fifth stage. Consequently, the coal begins to collapse, potentially resulting in a disaster and failure. It is, therefore, possible to accurately predict coal and rock dynamic failures and microcrack mechanisms by observing the subsequent sudden drop in the correlation dimension of the AE signals in response to different stages of loading.

## Introduction

The water–rock interaction is a hot issue in the field of engineering geology, notably in mining and geotechnical engineering^1–3^. This issue poses several geological hazards and engineering safety concerns, including dam instability, water inrush, and landslides^3–8^. The presence of water in underground environments, such as deep coal mines, leads to long-term deterioration of the mechanical properties of coal. Coal and rock masses undergo mechanical changes following water absorption due to changes in their structural components. The presence of water in a rock increases its plastic yield and softening degree and reduces its elastic modulus, stiffness, compressive strength, tensile strength, and brittleness^9–12^. Water content also significantly influences the rock fracture mechanics^13,14^. The effects of water must be fully considered to ensure the stability of underground mines. Consequently, the impact of water content on coal mechanical behavior must be considered when analyzing and monitoring these problems.

Acoustic emission (AE) is the immediate release of strain energy in the form of an elastic wave during material deformation^16–27^. The AE parameters and AE relationship to the mechanical failure process under compression have been extensively studied by researchers. By utilizing AE techniques, significant development has been made in understanding the progressive failure process of coal and rock. Researchers investigated coal and rock AE behavior under uniaxial compression, characterization of rock crack patterns under different loading rates, and coal AE fractal characteristics^28–33^. Water content has a significant impact on the AE characteristics of coal and rock, resulting in a decrease in elastic energy release and a reduction in the AE signal during loading^34^. Studies were conducted to investigate the effect of water content on failure patterns and AE features of rock with different water content. For this, uniaxial compression tests and numerical simulations with PFC_2D_ software were used to investigate the evolution of microcracks and failure patterns^15,35–43^. The results indicated that increasing water content within the rock significantly decreased the rock strength, Young's modulus, strain to peak strain ratio in the elastic deformation stage, the maximum energy of a single AE event, and average AE energy. Lin et al. studied the AE parameters during disc cutter-induced rock fragmentation processes under various water conditions^44^. Read et al. studied the evolution of microcracks in rocks under saturated conditions using AE data^45^. The results demonstrated that the varying characteristics of crack propagation in rocks could be characterized by the frequency of AE events combined with volume change. The AE parameters and P-wave velocity variations in the porous rock after microcrack closure were studied by Fortin et al.^46^. Zhou et al. have taken sandstone under different water contents as a research object, and the type I fracture mechanism and AE characteristics were investigated^47^. The results unveiled that increasing water content decreased fracture toughness (*KIc*). Zhou et al. also investigated the quasi-static fracture behavior of sandstone containing different water content. For this, notched semi-circular bending (NSCB) tests were conducted, and the cracking process and acoustic emission (AE) signals were recorded simultaneously^48^. Zhu et al. and Liu et al. have paid their efforts to investigate the frequency features of AE signals during loading on dry and water-saturated rocks. The outcomes revealed that the high-frequency AE signals were pointedly reduced by water content^26,49,50^. An Analytical damage model based on acoustic emissions was developed by Ali et al. for dry and saturated coal^51^. Recently, the researchers have introduced the multi-fractal theory to deconstruct the AE signals to reveal better the nonlinear and multi-scale features of the deterioration and fracture process of water-bearing rocks^52–54^. These studies have provided valuable insights into understanding rocks' AE characteristics and damage behaviors under different water conditions. Hitherto, the variation of AE, fractal characteristics, and mechanical parameters of natural and coal with different water content and soaking times is rarely reported.

This paper examines the fractal characteristics of coal with different water contents in order to evaluate the variation in AE characteristics and mechanical parameters during the failure process of the coal. Coal was taken as an example to prepare specimens with varying water contents based on different soaking times. Grassberger Procaccia (GP) algorithms were utilized to find AE fractal characteristics based on the theory of phase space reconstruction. Based on the results of the AE test, comprehensive data could then be synchronized with the results of the mechanical testing in order to determine the type and location of rupture as well as the failure process of the samples. Therefore, this technique can be successfully utilized to predict coal and rock dynamic failure and ensure safety for engineering projects.

## Methodology

### Preparation of the coal sample

**Figure 1:**
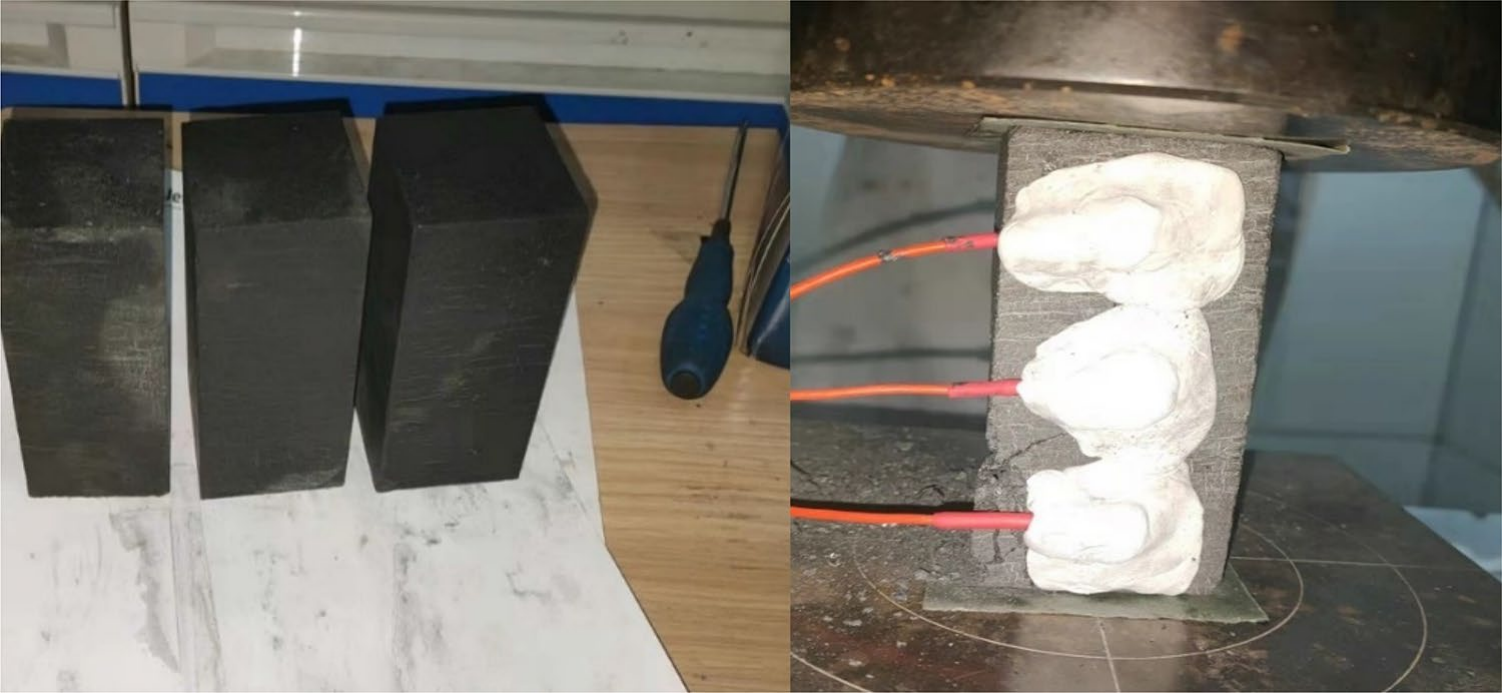
Coal samples from Shanxi province.

Test coal samples were obtained from the face of coal blocks in Shanxi Province. This sample's dimensions were determined using a vernier caliper, which is approximately 100 mm × 50 mm × 50 mm, and the weight was measured using an electronic balance. Figure 1 illustrates the sample used in the experiment. The coal samples were then placed in the water container. The coal samples were collected at various time intervals to determine the percentage of water content.

**Figure 2:**
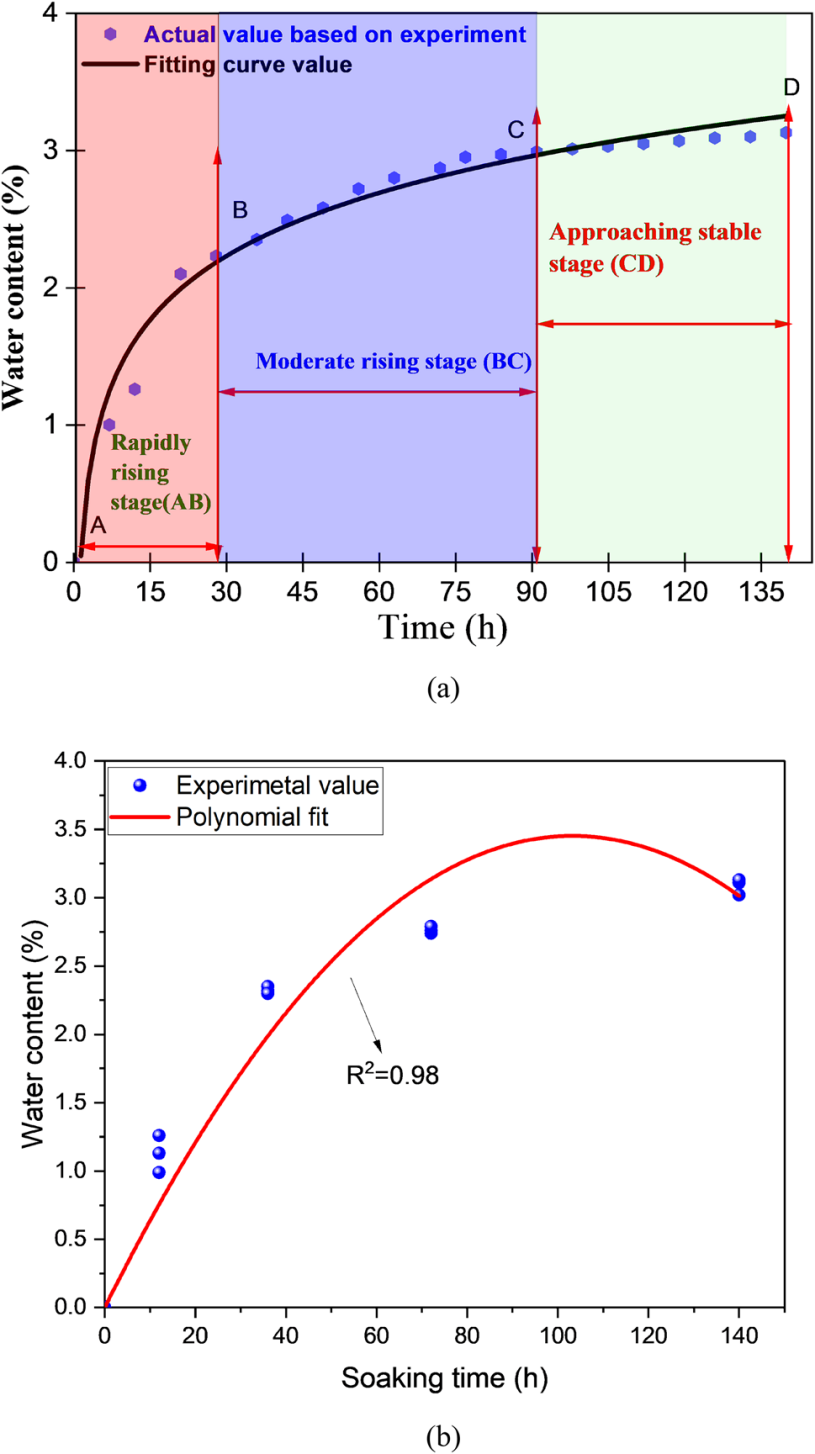
The relationship between soaking time and water content based on (**a**) saturation process (**b**) samples selected for experiment.

The water saturation curve is classified into three stages based on experimental saturation process results: Stage rapid rising is A to B (0–27 h), from B to C is the moderate rising stage (27–93 h), and C to D is the final approaching stage of stability (93–140 h) as shown in Fig. 2a. After 119 h, the water saturation level remained consistent at 2.913 percent by weight, suggesting that the coal was totally saturated. As a result, the samples were subsequently classified into five groups depending on their soaking time and water content: A, B, C, D, and E. Each group consists of 3 samples. Then, samples were stored in a completely covered container to prevent it from drying out or getting moist. The soaking times for coal samples remained 0 h, 12 h, 36 h, 72 h, and 140 h. The corresponding water content values absorbed are 0 percent, 0.991 percent, 2.136 percent, 2.76 percent and 3.109 percent, as shown in Fig. 2(b). The experimental results that differed from the average value of coal samples in each group were eliminated to reduce data dispersion. The elastic modulus, wave velocity, peak strength, and cumulative energy of coal at various water content are shown in Table 1.

**Table 1:** The different characteristics of coal samples.

Sample #	Wave velocity before soaking km/s	Wave velocity after soaking km/s	Soaking time (h)	Water content (%)	Elastic modulus (GPa)	UCS (MPa)
A1	2.31	3.31	140	3.02	0.820	8.05
A2	2.32	3.32	140	3.11	0.805	7.96
A3	2.34	3.33	140	3.13	0.801	7.93
B1	2.31	2.92	72	2.74	0.905	9.30
B2	2.30	2.91	72	2.76	0.890	8.76
B3	2.31	2.93	72	2.79	0.838	8.45
C1	2.32	2.84	36	2.32	0.940	9.46
C2	2.33	2.82	36	2.35	0.923	9.35
C3	2.31	2.83	36	2.30	0.954	10.2
D1	2.31	2.53	12	0.99	1.240	11.03
D2	2.31	2.52	12	1.13	1.230	10.77
D3	2.32	2.51	12	1.26	1.245	10.80
E1	2.34	2.34	0	0	1.310	14.23
E2	2.31	2.31	0	0	1.299	14.2
E3	2.30	2.30	0	0	1.287	14.19

### Experimental setup

**Figure 3:**
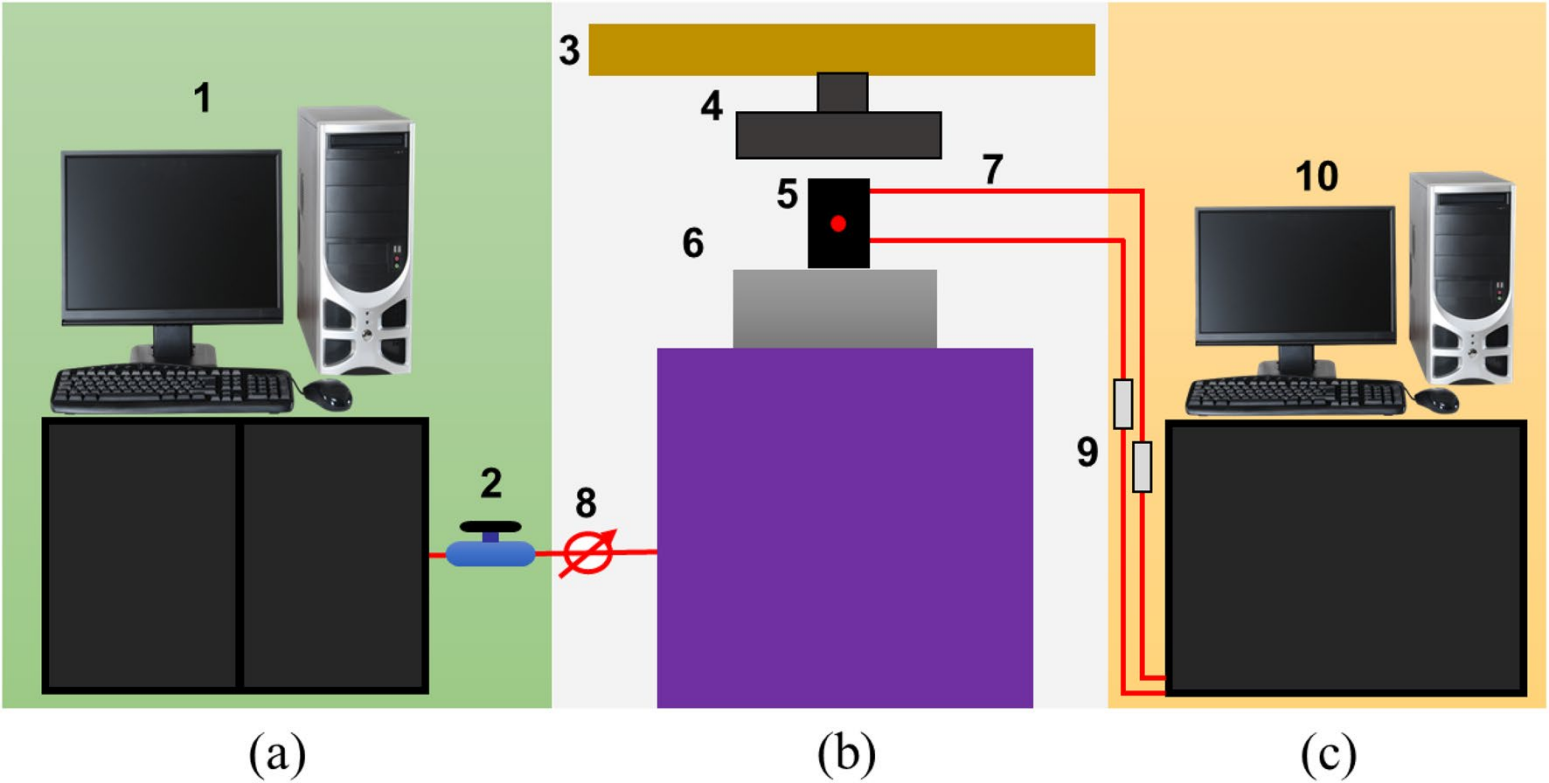
Systematic diagram experimental system. (**a**) Loading controls system includes (1) system for loading control (2) valve (**b**) AE signals collecting and a loading system consists of (3) Serve hydraulics press, (4) Press head, (5) coal sample, (6) AE Sensor, (7) connecting wires (8) Pressure gauge; (**c**) AE and EMR data processing and storage system comprises (9) pre-amplifiers, (10) system screen to display data and (11) AE monitoring device.

This system comprises a loading system, and a sensing and data-collecting system, as shown in Fig. 3.

#### Loading system

The composition of the system is shown in Fig. 3, and the tests carried out using this system are uniaxial compression tests (Uniform loading, cyclic loading). This experimentation equipment incorporated an axial loading subsystem and data acquisition system. The loading system (including the load displacement recording system) adopts the control system of Y4306, a 3000kN pressure testing machine. The system consists of a press host, loading control system, computer and power-test V33 composition of the control program. During the experiments, the pressure and displacement generated by the pressure head of the press are respectively transmitted to the loading control system with analog electrical signals through the pressure sensor and extensometer. The control box is converted into digital signals and transmitted to the computer. The computer software automatically collects and stores these data signals, and power-test V33. The software drives the press through the control box to realize the loading process automatically according to the manual instruction. During the experiment, the data sampling rate of force and displacement can reach 100 Hz, and the experimental curves of load time, deformation time, force deformation, and force displacement can be recorded and displayed in real time.

#### Data collection system

AE System (PCI-2) with eight channels was used to monitor and record the data. A detailed description of the parameters used in this experiment can be found in Table 2. The system includes an 18 bit A/D converter module, eight digital input/output channels, and two real-time data acquisition channels. The amplifier provides three output levels: 20 dB, 40 dB, and 60 dB. AE sensors are capable of responding to frequencies between 50 and 400 kHz, and are equipped with an electronic pre-amplifier of 20 dB. AE sensor signals are amplified by the preamplifier and transmitted to the conversion module. This data and the parameters of the digital signal are then stored in a buffer and are then transferred to a computer so that they can be further processed and displayed. Prior to the start of each experiment, sensors were attached to coal samples. We applied the load in displacement control mode at a loading rate of 0.200 mm/min. The loading system was initiated simultaneously with the AE monitoring system.

**Table 2:** Detailed description of the parameters AE System (PCI-2).

Number of sensors	Resonance frequency	Pre-amplification	Threshold level	Pre-trigger time	Sampling frequency	Recording length
8	300 kHz	40 dB	38 dB	256 points	2 MHz	2048 points

## Experimental results

### Stress–strain behavior of coal samples

**Figure 4:**
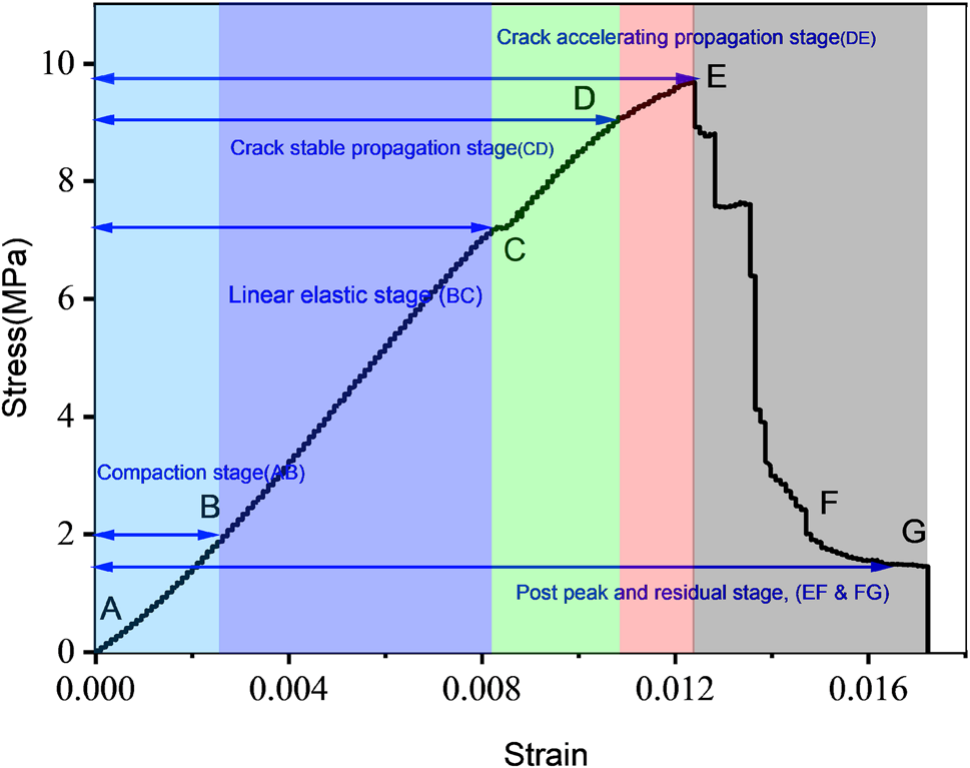
The coal's sample stress–strain curve.

According to a previous study, the stress–strain curve passes through several stages^4^. The stress–strain relationship for the entire uniaxial process on sample D1 is shown in Fig. 4. On the basis of this graph, it can be determined that the loading phase of the test is divided into five sections. The compaction stage, marked as AB, involves repeatedly squeezing the coal sample's original tiny fissures, thereby increasing its density. The stress–strain curve is said to be "convex-concave." After this stage, the stress–strain curve starts to converge, and the compressive stress is where compaction meets linear elasticity, and the increasing rate of strain is reduced over time.

The linear elastic stage (BC) depicts microscopic cracks, and weak joints are subjected to increased compression, but will not expand further as the stress is insufficient to induce new cracks or cause existing cracks to develop further. The stress–strain curve at this stage is a straight line with an inclined vertex, indicating that the rock deforms elastically with the applied load. Crack initiation stress marks the junction of the BC and CD stages. In Fig. 4, the crack stable propagation stage is represented by CD. At this stage, when the stress level in the rock exceeds the initial stress, the crack begins to widen. As a result of increased stress, new cracks continue to form in coal samples, deteriorating the mechanical properties of coal samples. A straight-line deviation in the stress–strain curve occurs due to loading, which initiates the stage of plastic deformation. This stage of accelerated crack propagation is referred to as DE, and it involves the propagation, development, and coalescence of cracks in coal, resulting in the formation of fracture networks. Macro fractures eventually penetrate, releasing energy and expanding the volume. In light of the qualitative change in the microcrack development process, the fracture continues to develop even when the stress remains constant. As a result, the coal sample bears a maximum load after this step has been completed. In the post-peak and residual stages (EF and FG), the stress decreases dramatically after the peak point of the stress–strain curve and remains constant as the strain increases (residual stress). The coal sample still has some bearing capability, and the deformation is primarily due to fractured coal dislocation sliding. The residual stress can be determined using the stress–strain curve, which is the constant value after the peak value of the stress–strain curve.

**Figure 5:**
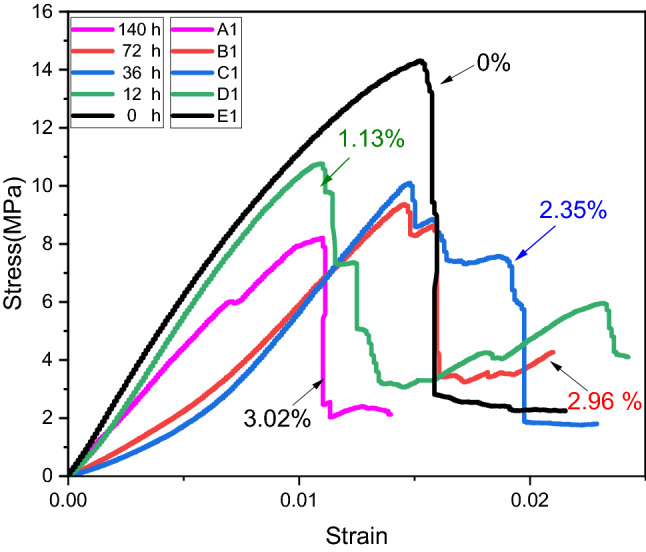
The stress strain curves of coal sample with different water content.

**Figure 6:**
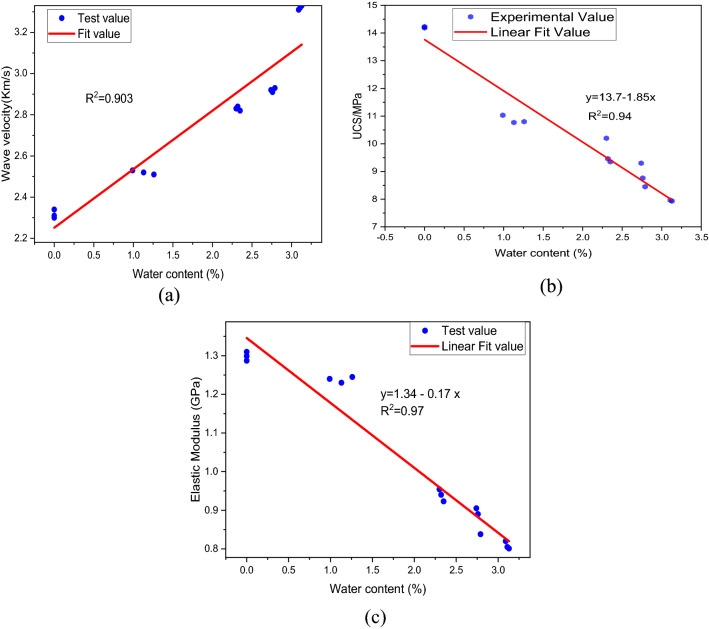
Properties of coal with different soaking time (**a**) wave velocity, (**b**) UCS, (**c**) elastic modulus.

Figure 5 illustrates the stress–strain curves of coal samples based on varying water content and soaking time. It is important to note that, despite the variation in stress levels of coal samples at various stages, all stress–strain curves have passed through the five stages described above. Furthermore, the peak stress associated with UCS varies with water content and soaking time. A study was conducted in which UCS, elastic modulus, and wave velocity of coal were analyzed in relation to water content. According to Fig. 6, the measured values for each set of samples are represented by a solid line, which represents the equation of linear fit as follows:

1 $$y=13.7-1.85x$$

Equation (1) can be written as

2 $${\sigma }_{u}=13.7-1.85w$$

where ${\sigma }_{u}$ is the UCS and is measured in MPa, and $w$ shows the water content. The UCS of samples with varying water contents is lower than that of natural samples, and UCS gradually decreases as the water content increases. Accordingly, increasing the water content of coal samples results in a decrease in UCS, and water content appears to have a significant impact on UCS. For instance, it is evident in Fig. 5, that the UCS of a sample containing 0% water is 14.30 MPa, whereas the UCS of a sample containing 3.02% water is 8.16 MPa and the loss rate of UCS is 42.93%. Furthermore, the sample with 0% water content showed a sudden drop after the peak stress, demonstrating that coal without water has a significant brittle failure characteristic. In contrast, the yielding characteristics of samples with high water content are particularly evident at the third and fourth stage of loading. It can be seen from Fig. 5 that each sample exhibits a unique stress and strain corresponding to the peak stress. Furthermore, since each specimen's stress level varies during the elastic deformation stage, it is necessary to examine the EM variation law with regard to water content. “Elastic modulus is the ratio of stress, below the proportional limit, to the corresponding strain”. It is a critical parameter affecting the performance of engineering materials. Elastic modulus (EM) is used to determine the resistance of materials to deformation on a macroscopic level. In elastic deformation stage, the slope of stress–strain curve is used to calculate EM as depicted in Fig. 4.

Figure 6 show the relationship of water content with wave velocity, UCS and elastic modulus (EM) respectively. Figure 6a show the linear fitting of wave velocity of coal with moisture content. The fitting curve show a good correlation with R^2^ value of 0.903. This demonstrate that the wave velocity in the coal mass gradually increases as the water content of the coal mass increases. The reason for this is that sound waves travel faster in water than in air and in coal masses with increased moisture content, water occupies a greater volume in the mass, while air occupies a smaller volume. Figure 6b show that the peak stress (UCS) of coal with varying water content is negatively correlated with water content with R^2^ value of 0.94. This indicate that the UCS of coal greatly influenced by water content and decreased with increasing water content. The reason for this is that water molecules gradually intrude into the coal samples, filling pores, and tend to provide, softening, and disintegrating effects, which eventually lead to destruction when comes under external load. The peak stress has a minimum value at maximum water content as shown in Fig. 6b. The EM value of a water-containing coal sample is less than that of a natural coal sample, showing that the presence of water in the coal sample can diminish the EM value. According to the fitting curve depicted in Fig. 6c, the values of EM declines linearly with the soaking time indicating a good negative correlation with R^2^ value of 0.97. Compared to the samples containing water content, the EM of natural samples is the greatest indicating their strength and resistance to deformation. Additionally, while there is some inaccuracy amongst samples with the same soaking period and water content, the average value of EM decrease progressively as the water content increases.

Energy dissipation is the most fundamental aspect of rock deformation and failure, reflecting the resulting development of new internal cracks, which weaken and ultimately disappear the strength of the material. According to the viewpoint of energy, the essence of the change of the physical state of matter is the conversion of energy, so rock failure can be regarded as state instability driven by energy.

Liu et al.^34^ state in their study that the equation of strain energy evolution law under uniaxial compression can be calculated as follows;

3 $$U = \int_{0}^{\varepsilon } \sigma d\varepsilon$$

where *U* is the total energy input by the press, $\sigma$ is the stress, and $\varepsilon$ is the strain.

Elastic strain energy *U*_e_ picked up by the rock during the loading process, which can be calculated using Eq. (4).

4 $$U_{{\text{e}}} = \frac{{\sigma^{2} }}{2E}$$

where *E* is the elastic modulus, and *U*_*e*_ is the elastic strain energy. Therefore, based on first law of thermodynamics, the dissipation energy U_d_ is calculated by Eq. (5)

5 $$U_{d} = U - U_{e}$$

**Figure 7:**
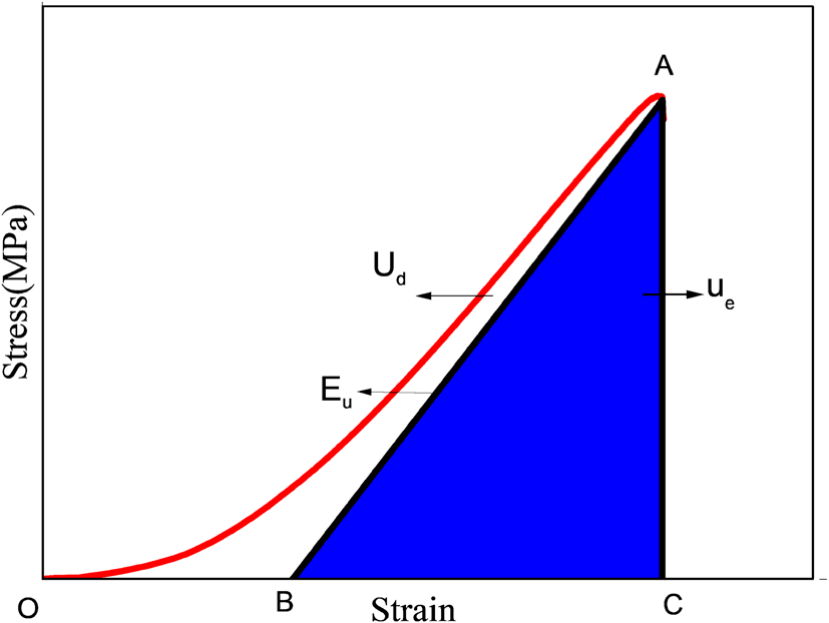
In stress–strain curve, analysis of the relationship between dissipation strain energy and elastic strain energy.

The strain energies of coal under the loading process are shown in Fig. 7.

### AE responses and dissipation energy during the loading process

**Figure 8:**
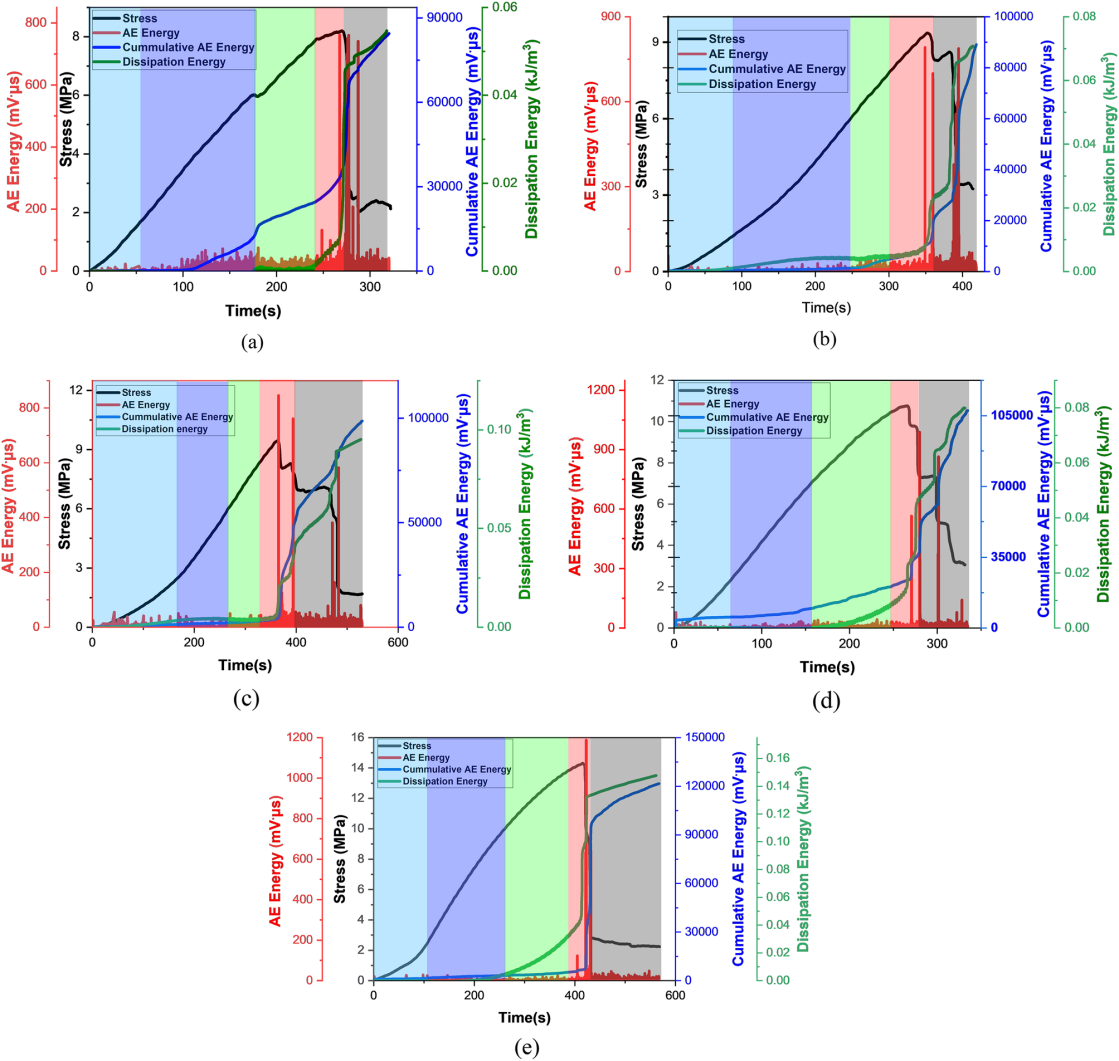
Illustration of the relationship between acoustic emission energy, cumulative and dissipation energy over time of group 1 samples, (**a**–**e**) respectively.

In this study, the AE energy, AE cumulative energy, and dissipation energy of coal samples with different water content were analyzed. Figure 8. illustrates that coal samples with varying water content and soaking time under uniaxial compression have different AE energy, AE cumulative energy, dissipation energy, and stress time curves.

The findings indicate that AE energy and AE cumulative energy characteristics vary depending on the stage under stress, as well as AE characteristics that vary based on the water content of coal samples. Figure 8. illustrates a good correlation between AE energy and stress at all stages of the process. The loading process has five stages based on stress/strain curve. In order to determine the variation law of AE at different stages, the features of AE are examined. As can be seen in Fig. 8, all samples exhibit almost similar trends in AE under loading conditions.

In order to understand the behavior of AE parameters under the applied load, the AE characteristics were studied in accordance with the five stages, using D1 samples as an example. The first stage is the compaction stage, where the closing of coal cracks due to stress, resulting in a very limited number of AE events. The second stage is the linear elastic stage, where the coal's distortion enters the elastic deformation stage after it has been compressed. As a result, no new cracks are formed, the number of AE events is significantly reduced, and the AE energy is kept to a minimum. Stage 3 is the stable crack propagation-SCPS-stage, where the stress reached the point of crack initiation, the AE energy increased dramatically, indicating new cracks generated and propagated. Stage 4 is referred to as "crack accelerated propagation stage" where, the cracks continue to expand and consolidate until eventually a fracture network formed, resulted in macro cracks. After the macro cracks were coupled, large quantities of elastic energy were released, which had been stored during the primary phases of the sample compression, leading to very active acoustic emission events. As a result, the acoustic emission energy reached its maximum instantly. As a result of this, there is also a sudden change in the dissipation energy and the cumulative AE energy. Stage 5 is the "post peak, and residual phases", where the stress lowers, and the acoustic emission energy drops dramatically after the coal sample is failed. However, residual stress results in some residual strength in the coal sample, resulting in many secondary fractures that form and expand. There is a slight increase in AE energy rate prior to failure. The cumulative energy and dissipation energy also varied accordingly.

Although the AE characteristics of samples with varying water contents are almost identical throughout the loading process, some differences are evident when samples fail. The instantaneous AE energy rate of samples A_1_, B_1_, and C_1_ is around 761 mV μs,792 mV μs, and 846 mV μs, and samples D1 and E are 945 mV μs, 1088 mV μs, respectively, when the final failure occurs. The large difference in acoustic emission energy rate during coal failure may be connected to the sample's failure mechanism. However, it is concluded that the AE energy decreases with the increase in water content. This could be linked to the failure stress drop mode. The stress of samples A_1_ and B_1_ approaches the residual stress after one or two abrupt dips, as illustrated in Fig. 8a,b, exhibiting the instantaneous release of the elastic energy accumulated in the sample. After two abrupt drops in stress, sample D_1_ reaches its minimal value, demonstrating that the stored elastic energy was not instantaneously released but rather due to many fractures. As a result, D_1_'s maximum AE energy rate is comparable to samples A_1_ and B_1_.

Furthermore, the stress of sample D_1_ drops to zero, as shown in Fig. 8d, quickly after the peak, although the maximum AE energy rate is substantially lower than that of the other samples. This is because coalescence occurs from the side of the sample, and neither sample has any cracks on the front surface. When AE is not present, the specimen's eventual failure rate is lower than other specimens. The cumulative energy of A_1_, B_1_, C_1_, D_1,_ and E_1_ are 87,025 mV μs, 89,154 mV μs, 100,020 mV.μs,105,630 mV μs, and 121,005 mV μs, respectively, Indicating that the cumulative energy of AE is also affected by water content and concluding that the greater the water content, the lower the cumulative energy of AE.

Under varying water contents, the dissipation energy and cumulative energy curves demonstrate a consistent trend. Figure 8 shows that the dissipation and AE cumulative energy curves show a linear trend in the compaction and linear elastic stages, and a slight stable growth at the stable crack propagation and crack propagation accelerating stages. However, an abrupt rise occurs when the loading approaches peak, which can be used to predict failure. As shown in Fig. 8a,e, the coal's cumulative energy and dissipation energy curves increased dramatically when it was loaded from a linear elastic state to a stable crack propagating stage. In addition, the dissipation energy value increased with increasing moisture content at peak loading stage, indicating that a significant amount of energy was dissipated in plastic deformation and crack development.

**Figure 9:**
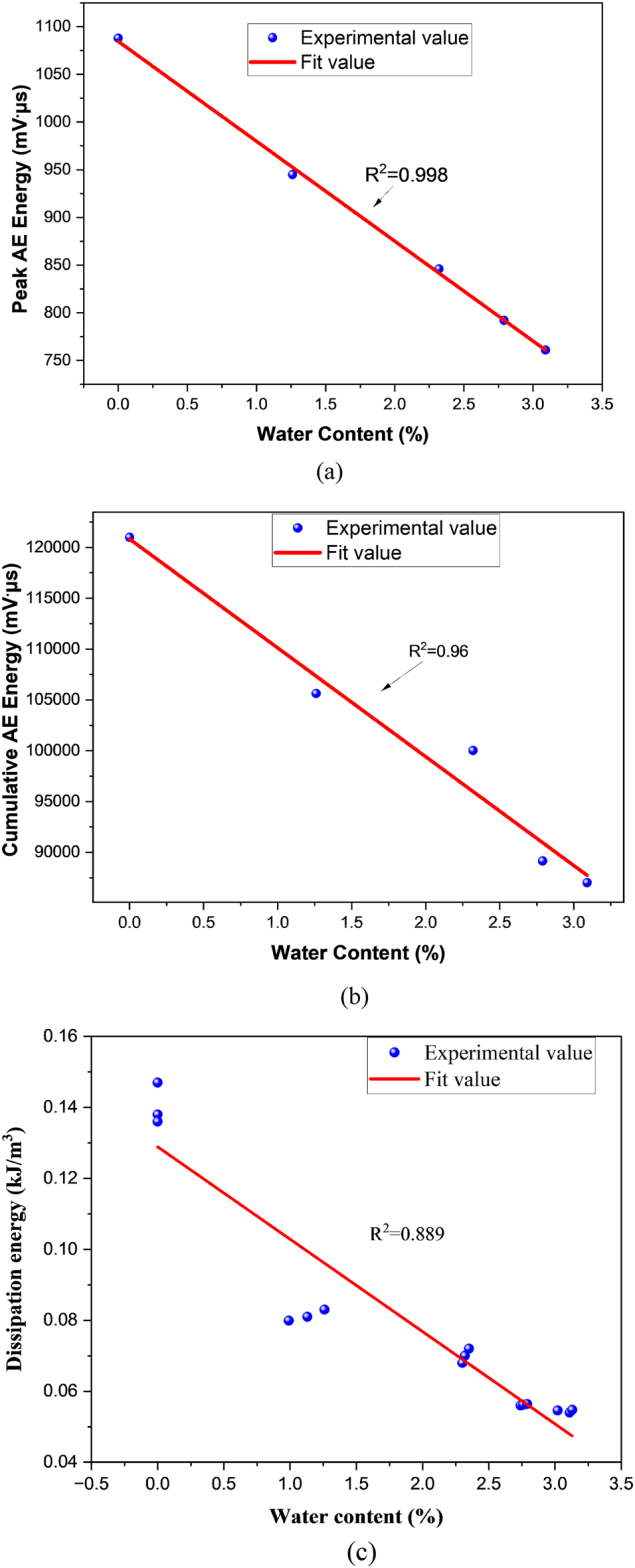
The correlation of water content with (**a**) peak AE energy (**b**) cumulative AE energy (**c**) dissipation energy.

The peak dissipation energy values of A_1_, B_1_, C_1_, D_1,_ and E_1_ are 0.0546 kJ/m^3^, 0.05600 kJ/m^3^, 0.070 kJ/m^3^,0.0799 kJ/m^3^ and 0.147 kJ/m^3^, indicating that the dissipation energy is also affected by water content and concluded that the greater the water content, the lesser is the dissipation energy. There is a negative linear correlation between peak AE energy and cumulative AE energy during the loading process with that of water content, as shown in Fig. 9.

### Acoustic emission fractal characteristics

#### Algorithms of G-P

The Grassberger Procaccia (G-P) method is generally applicable for computing a series of numerical values using correlation dimension^56^. Considering the energy of acoustic emission as a set of numbers in the process of rock uniaxial compression deformation and failure. The dimensioned series can be stated as:

6 $$X = \{ x_{1} ,x_{2} \ldots ,x_{n} \}$$

On the basis of X (m < n), the phase space m-dimensional can be constructed. The first vector, ${X}_{1}$, is built by taking m numbers out of X continuously:

7 $$X_{1} = \{ x_{1} ,x_{2} \ldots ,x_{m} \}$$

Get the second vector by moving one number right.

8 $$X_{2} = \{ x_{1} ,x_{2} \ldots ,x_{m + 1} \}$$

You may obtain vectors N = n-m + 1 and define the correlation function (C) associated with it as follows:

9 $$C(r) = \frac{1}{{N^{2} }}\sum\limits_{i = 1}^{N} {\sum\limits_{j = 1}^{N} {H\left[ {r - \left| {X_{i} - X_{j} } \right|} \right]} }$$

H represents the Heaviside function which is as follows:

10 $$H(u) = \left\{ \begin{gathered} 0, u < 0 \hfill \\ 1, u \ge 0 \hfill \\ \end{gathered} \right.$$

Scale function is denoted by r. The following formula is used to determine the r value in order to avoid dispersion:

11 $$r = k\frac{1}{{N^{2} }}\sum\limits_{i = 1}^{N} {\sum\limits_{j = 1}^{N} {\left| {X_{i} - X_{j} } \right|} }$$

Here $K$ denotes the factor of scale. Researchers found that AE fractal characteristics are not obvious if the k value is less than 0.1. For this reason, the range of k values was kept from 0.2 to 1.4.

There is a $C(r)$ -correlation function, which corresponds to a particular $r$. The points $(lgr, lgC(r))$ are presented and fitted using double-coordinated logarithms. In the case where the fitted outcome is a straight line, this signifies that the acoustic emission energy sequence is fractal, with D representing the straight line, and can be expressed as:

12 $$D = {{\ln C(r)} \mathord{\left/ {\vphantom {{\ln C(r)} {\ln r}}} \right. \kern-0pt} {\ln r}}$$

The physical significance of the AE series is known from the correlation dimension that increases the correlation dimension D value, the greater the irregular nature of the fractures, more than half of which are micro-fractures in the coal sample.

#### Phase space (m) determination

**Figure 10:**
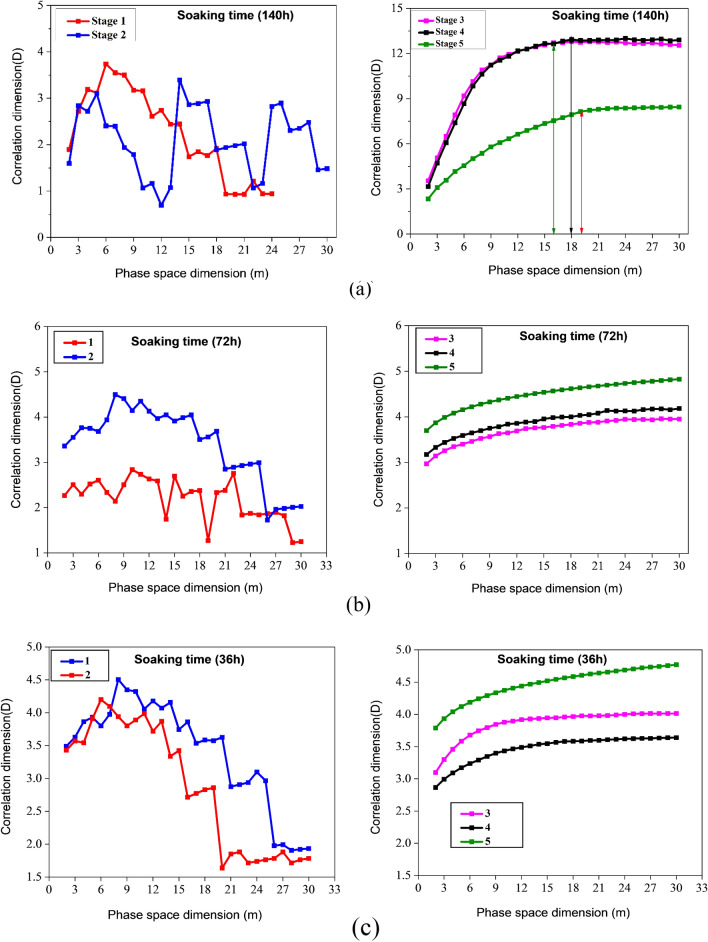

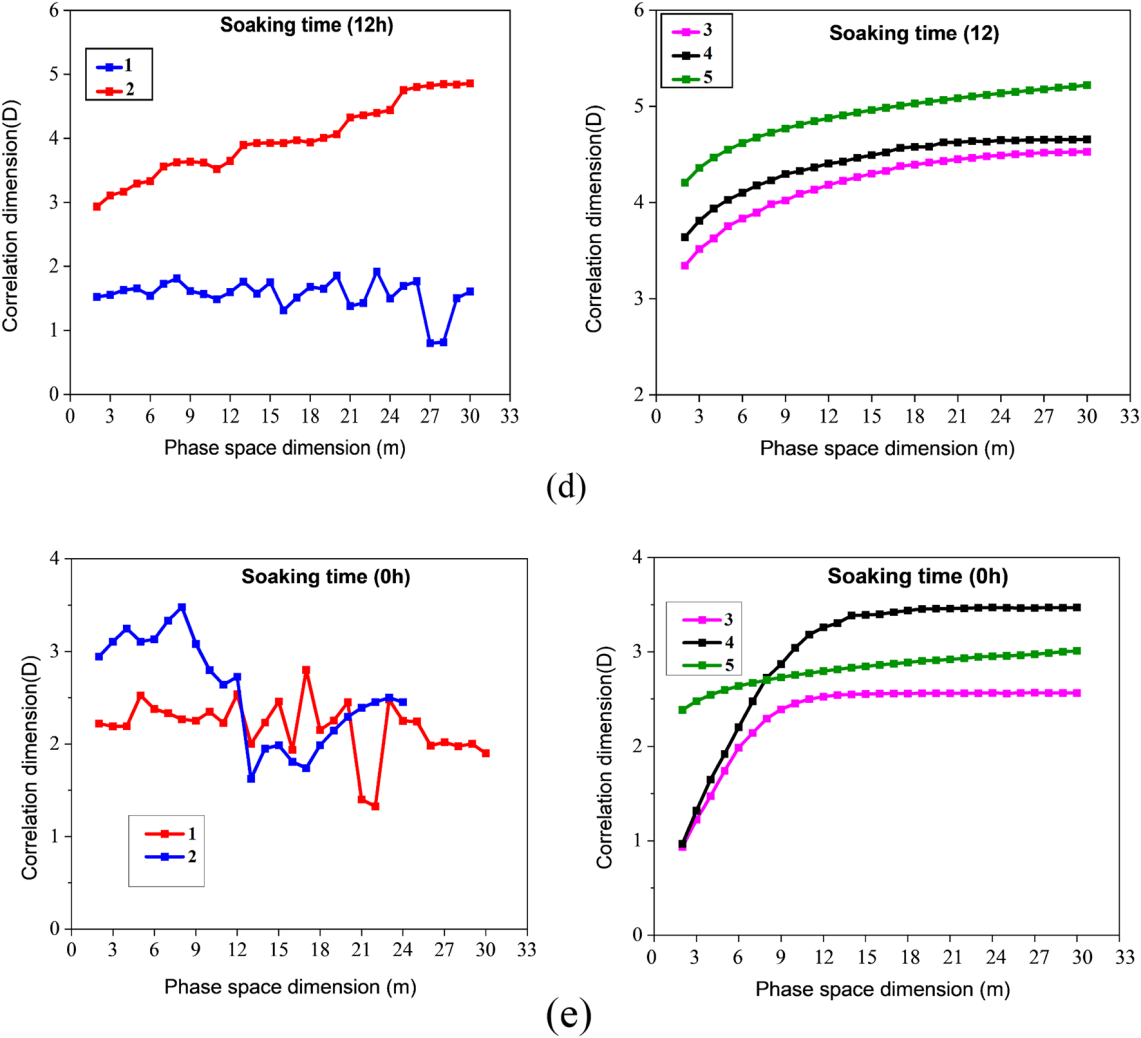
Coal sample AE signal phase space and correlation dimensions with various water content and soaking time during uniaxial compression.

A crucial factor in determining the value of the correlation dimension (*D*) is the phase space dimension (*m*). Figure 10 illustrates how $D$ varies with the phase space dimension $m$. Research indicates that $D$ value increases in proportion to the $m$ value until $m$ reaches a specific value, at which point $D$ remains constant. This is why the initial value of D was selected at a stable stage to study $m$ further. For instance, the third, fourth, and fifth stages of AE fractal analysis of sample A (140 h), and $D$ become stable at 16, 18, and 19, respectively. Therefore, 16, 18, and 19 are selected as the phase space dimensions for those three stages. Using the same approach, we determine the phase space dimensions for the remaining specimens in stages 3, 4, and 5 using Fig. 10b–e, as mentioned in Table 3. Furthermore, D varies significantly from the first to the second stage and doesn't have a constant value, the AE energy in these two stages is believed not to have any fractal properties. The third, fourth, and fifth stages require Eq. (12) verification to see whether the AE energy exhibits fractal properties.

**Table 3:** Dimensions of phase spaces in different stages of a specimen.

Phase	Sample A1	Sample B1	Sample C1	Sample D1	Sample E1
3	15	21	13	21	16
4	17	21	15	21	19
5	18	19	19	17	21

#### Correlation dimensions of AE energy

**Figure 11:**
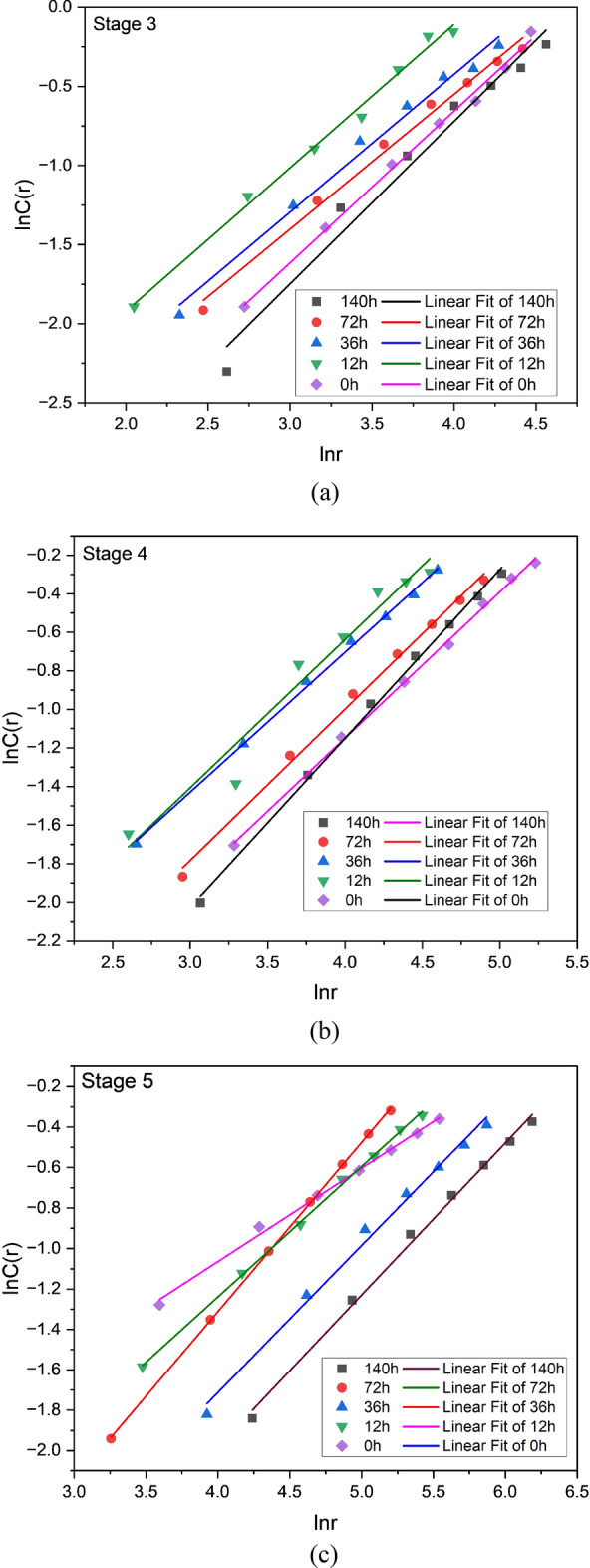
Illustration of the logarithmic relationship between acoustic emission energy and coal sample with various water content.

lnC(r) and ln(r) function of water content for specimens subjected to varying stages of loading are plotted in Fig. 11. As illustrated in Table 4, each data point set is matched with a straight line, and the fitting correlation coefficient is calculated as R^2^. Consequently, D is determined using Eq. (12). Table 4 indicates that Phases 3, 4, and 5 demonstrate R^2^ values greater than 0.90 in all specimens, with the majority exceeding 0.96. As a result, it can be assumed to be linearly connected to D, implying that the AE energies of all samples in phases 3, 4, and 5 exhibit fractal properties. Changes in D can represent the evolution of cracks in coal, depending on the nonlinear characteristic function of AE energy. As a result, D is a correlation dimension that's useful for predicting rock and coal internal mechanical behaviors and structural degradation.

**Table 4:** The R^2^ and D for different phases of samples.

Stage	Sample A1		Sample B1		Sample C1		Sample D1		Sample E1	
	R^2^	D	R^2^	D	R^2^	D	R^2^	D	R^2^	D
3	0.988	3.350	0.990	0.853	0.988	0.871	0.968	1.028	0.955	0.959
4	0.987	2.870	0.995	0.785	0.998	0.726	0.997	0.876	0.948	0.759
5	0.901	0.981	0.959	0.836	0.991	0.728	0.995	0.751	0.925	0.461

In accordance with the research presented above, AE energy lacks fractal qualities in the first and second stages but exhibits prominent fractal characteristics in the third stage. From the AE graphs depicted in Fig. 8, each sample has a distinct pattern in its AE histograms. As the sample is lacking in few cracks, AE does not frequently occur during the compaction stage. Therefore, the AE energy displays the scattering distribution as pulses. After entering an elastic deformation stage, there aren't any new fractures, so the number of AE events drops even further. Thus, the AE energy is low in stages 1 and 2 during compression, and the time domain exhibits a paroxysmal distribution, so there is no fractal pattern. During the third deformation stage, the coal sample reached the original stress level, and the internal fracture expanded. The stress level rises as new cracks appear, and the AE energy remains high. In addition, the AE energy begins to display fractal characteristics, and the development starts to have fractal characteristics. During testing sample has reached the fourth stage in its deformation, crack propagation and coalescence take place, and the number of acoustic emissions increases significantly. As soon as stress reaches its peak, the breaking of the sample causes a maximum AE energy. In coal samples, the damage progresses from disorder to order, from whole to local, and the major fault zone is gradually developed during the crack coalescence process, resulting in a stagnation of the $D$ value. The stress reduces and varies towards the residual stress once the sample deformation enters the fifth stage, while the AE energy remains around a specific number, though more significant than before fracture. It has something to do with the creation of a lot of fault planes and the major staggered sliding between them. However, in both cases, the fourth and fifth stages' correlation dimension $D$ is lower than the third stage's, indicating that the fractal dimension approaching instability or fracture and final primary failure is lower than the "stable stage." The stable stage is described as when a coal sample's internal crack begins to expand slowly and steadily but not to the point of apparent fracture, comparable to the third stage of coal deformation. The stress in the "stable stage" may be 50–70% of the peak stress, depending on the relevant dimensions and stress levels. It is allowed in the coal mining process for no more than 80% of the coal's strength should be imposed on coal and rock mass.

AE energy does not have fractal characteristics in the first loading stage, according to the variation law of $D$ discussed at different phases (the first and second stages). The acoustic emission energy reveals evident fractal characteristics after entering the third stage, and the correlation dimension may be determined. The fractal dimension began to drop or initially decrease and then climbed, but the fourth and fifth stages' D was lower than the third. As a result, a set of evolution models of AE energy correlation dimension are summed throughout the complete compression process of coal samples; namely, the correlation dimension "does not appear—continually lowers (or decreases and then increases)." Although the D value of the sample in each stage is different, the changing trend is mainly similar, showing that a change of D value may be utilized to expose the internal damage of the sample, according to the fractal characteristics of acoustic emission energy obtained above.

## Discussion

According to the findings, the presence of water in coal samples reduces the EM and UCS. This study's mechanical behavior can be compared to that of prior investigations^57^. A major finding of the study is that the UCS of coal decreases as the water concentration increases, as demonstrated by Hongru Li et al. who also examined the effect of water content on the mechanical characteristics of rock specimens during uniaxial compression^58^. In this study, we investigate the impact of water content on the mechanical properties, the acoustic emission response, and the fractal characteristics of coal samples, and provide practical recommendations as a result. Based on the results, we conclude that the amount of water in a rock has a profound effect on its mechanical behavior and AE signals. As the water content increases, the UCS and EM decrease, whereas peak strain and energy dissipation increase. However, the research was limited to samples with low moisture content and short periods of soaking. In a study conducted by Ali et al., EM and UCS of saturated coal samples gradually decrease with increasing water content^4^. Laboratory and theoretical analyses have previously confirmed the mechanical properties of coal specimens under uniaxial compression. Researchers may find the results to be useful in solving problems related to underground engineering, such as those associated with water-rich coal environments.

Despite the fact that coal samples with varying water content have different correlation dimensions, D is essentially the same in all samples, suggesting that the change in D can be used as an indicator of the level of damage in coal. There were no fractal characteristics to be observed during the first and second stages of the AE energy. However, the third stage clearly exhibited fractal characteristics. At the fourth stage of the process, the process abruptly declined, and at the fifth stage, there was a slight increase and decrease. Xu et al. suggested that changes in the correlation dimension are indicators of crack emergence and evolution in rock samples^59–64^. According to Li et al., when coal under loading reaches a specific stress level, the correlation dimension of AE shows a downward trend, and the failure rate of coal reflects the fractal law as coal failure progresses. It's because of decreasing correlation and increasing order^65^. Scholars concluded from the findings that the variation in AE energy rate helps predict monitoring and provides an early signal for coal failure because AE energy rate varies due to coal's internal damages. More research is being done to improve the AE technology's effectiveness in coal and rock mass monitoring. Using the static expansion fracture loading method, Li and Zhou investigated the AE characteristics of the entire process of failure in a mine situated at Wushan. According to the AE domain sequence, they split the fault into four stages: (1) the beginning stage; (2) the stage of violence; (3) the descent phase; and (4) the quiet phase^66,67^. Between the violent stage of very active AE signals and rock fracture, there is a lowering stage and a calm stage, showing that the acoustic emission activity progressively declines after the peak and eventually subsides, named the "calm stage." This peaceful interval does not indicate that the coal and rock mass is stable but rather that a structural failure is imminent. Li et al. also hypothesized that a sudden drop in AE's or a comparatively quiet interval preceding roof collapse, spalling, or rock burst can be observed in the field^67^. Vl et al. used laboratory experiments to corroborate the "calm period" preceding rock failure. The researchers examined this phenomena's physical process and mechanism^29^.

On the other hand, taking quiet time as a sign of impending coal or rock mass destruction has caused complications in field monitoring. The researchers discovered that the AE calm period occurs before the outburst of coal but that the quiet period in the time domain does not fully correspond to these disasters. In other words, particular coal and dynamic rock disasters may not have a quiet phase before them, and not all calm periods have a dynamic event after them, as our experimental data show. Samples D_1_ and E_1_ have a clear calm phase before failure, as illustrated in Fig. 8d,e, whereas other samples do not. Other AE indices or analytical methods should be integrated with AE energy and "silent period" to monitor coal mass stability using acoustic emission technology successfully.

According to the above analysis, the correlation dimension of AE energy can be utilized to monitor coal's mass stability during mining. In other words, AE data is gathered and processed using fractal analysis during tunnel excavation or coal mining. Initially, the coal did not exhibit fractal characteristics; however, as soon as fractal characteristics appear, the coal and rock mass enter a phase of crack propagation. In the event that the correlation dimension suddenly decreased, the crack accelerating propagation stage would emerge, and the coal would degrade, possibly leading to a major coal failure. Consequently, prompt action must be taken to monitor and study the coal and rock mass in this area in order to prevent tragic consequences.

## Conclusions

In this paper, we study the characteristics of coal samples with different water content. The failure characteristics of the coal samples with different water content is monitored and analyzed by AE during the loading process.

Based on the experimental results, we categorized the water saturation curve into three stages: the rapid rising stage is A to B (0–27 h), from B to C is a moderate rising stage (27–93 h), and C to D is the final approaching stable stage (93–140 h). The water saturation level remained constant at 3.13% by weight after 119 h, indicating that the coal had been saturated to a complete extent.

Water content in coal specimens has a significant impact on mechanical properties. As the amount of water in the coal sample increases, the UCS, dissipation energy, peak stress, and elastic modulus of the coal sample decrease. In contrast, the wave velocity of coal samples increases as the water content increases. The dissipation energy and AE cumulative energy experienced a sudden change and can be used as precursors.

The AE energy, cumulative energy and dissipation energy response of coal samples having different water content were correlated with stress throughout the loading process, but the peak values varied when failure occurred. The peak AE value of a natural coal sample is 40% higher than the peak value of a fully saturated coal sample after 140 h. Generally, the lower the water content, the higher the peak value of acoustic emission energy will be. Additionally, the cumulative AE energy of natural samples is higher than that of samples with different water content.

It is apparent that fractal characteristics were absent in the first and second stages, whereas fractal characteristics were clearly evident in the third stage. The correlation dimension rapidly decreases as the strength reaches its maximum in the fourth stage, indicating the presence of macrocracks; however, the fractal dimensions continue to decrease or increase slightly in the fifth stage. As a result, the coal begins to collapse, potentially resulting in a coal disaster. It is possible to identify early warning signs by observing the sudden variation in the correlation dimension.

## Data Availability

The datasets used and/or analyzed during the current study available from the corresponding author on reasonable request.
